# Progressive Enrichment of Stemness Features and Tumor Stromal Alterations in Multistep Hepatocarcinogenesis

**DOI:** 10.1371/journal.pone.0170465

**Published:** 2017-01-23

**Authors:** Jeong Eun Yoo, Young-Joo Kim, Hyungjin Rhee, Haeryoung Kim, Ei Yong Ahn, Jin Sub Choi, Massimo Roncalli, Young Nyun Park

**Affiliations:** 1 Department of Pathology, Yonsei University College of Medicine, Seoul, Republic of Korea; 2 Integrated Genomic Research Center for Metabolic Regulation, Yonsei University College of Medicine, Seoul, Republic of Korea; 3 Severance Biomedical Science Institute, Yonsei University College of Medicine, Seoul, Korea; 4 Natural Products Research Center, Korea Institute of Science and Technology (KIST), Gangneung, Gangwon-do, Korea; 5 Brain Korea 21 PLUS Project for Medical Science, Yonsei University College of Medicine, Seoul, Korea; 6 Department of Pathology, Seoul National University Bundang Hospital, Seoul National University College of Medicine, Seongnam, South Republic of Korea; 7 Department of Surgery, Yonsei Liver Cancer Special clinic, Yonsei University College of Medicine, Seoul, Korea; 8 Department of Pathology, Humanitas Clinical and Research Hospital and Hunimed University of Rozzano, Milan, Italy; Kanazawa University Hospital, JAPAN

## Abstract

Cancer stem cells (CSCs), a subset of tumor cells, contribute to an aggressive biological behavior, which is also affected by the tumor stroma. Despite the role of CSCs and the tumor stroma in hepatocellular carcinoma (HCC), features of stemness have not yet been studied in relation to tumor stromal alterations in multistep hepatocarcinogenesis. We investigated the expression status of stemness markers and tumor stromal changes in B viral carcinogenesis, which is the main etiology of HCC in Asia. Stemness features of tumoral hepatocytes (EpCAM, K19, Oct3/4, c-KIT, c-MET, and CD133), and tumor stromal cells expressing α-smooth muscle actin (α-SMA), CD68, CD163, and IL-6 were analyzed in 36 low grade dysplastic nodules (DNs), 48 high grade DNs, 30 early HCCs (eHCCs), and 51 progressed HCCs (pHCCs) by immunohistochemistry or real-time PCR. Stemness features (EpCAM and K19 in particular) were progressively acquired during hepatocarcinogenesis in combination with enrichment of stromal cells (CAFs, TAMs, IL-6+ cells). Stemness features were seen sporadically in DNs, more consistent in eHCCs, and peaked in pHCCs. Likewise, stromal cells were discernable in DNs, showed up as consistent cell densities in eHCCs and peaked in pHCCs. The stemness features and tumor stromal alterations also peaked in less differentiated or larger HCCs. In conclusion, progression of B viral multistep hepatocarcinogenesis is characterized by an enrichment of stemness features of neoplastic hepatocytes and a parallel alteration of the tumor stroma. The modulation of neoplastic hepatocytes and stromal cells was at low levels in precancerous lesions (DNs), consistently increased in incipient cancer (eHCCs) and peaked in pHCCs. Thus, in B viral hepatocarcinogenesis, interactions between CSCs and the tumor stroma, although starting early, seem to play a major role in tumor progression.

## Introduction

Cancer stem cells (CSCs), a subset of tumor cells, exhibit the ability to self-renew and initiate (promote) tumor formation, and they also contribute to rapid tumor growth and chemoresistance [1]. Reportedly, hepatocellular carcinomas (HCCs) with stemness features, which express hepatic stem cell (HSC) markers, such as keratin19 (K19), CD133, or epithelial cell adhesion molecule (EpCAM), are associated with a higher incidence of vascular invasion and poorer prognosis, compared to HCCs lacking these markers [2, 3].

The biological behavior of tumors is known to be affected not only by the tumor cells themselves but also by their interactions with the adjacent stroma [4]. The tumor stroma consists of several cellular components, including cancer-associated fibroblasts (CAFs) (also known as myofibroblasts), tumor-associated macrophages (TAMs), cell signaling molecules, extracellular matrix proteins, and blood vessels [5]. CAFs have been reported to promote tumor growth and invasion and to stimulate angiogenesis, and associations between CAFs and aggressive biological behavior, poor prognosis, and chemoresistance have been demonstrated in various malignancies including HCC [6]. TAMs are responsible for mediating the wound healing processes via extracellular matrix remodeling, angiogenesis, and immunosuppression [7], and they were reported to be correlated with worse prognosis in various cancers, including HCC [8]. Additionally, IL-6, a multifunctional inflammatory cytokine produced by CAFs and TAMs, has also been shown to play important roles in tumor progression [9].

Increasing evidence suggests that human hepatocarcinogenesis is a multistep process, progressing from low grade dysplastic nodules (LGDNs), high grade DNs (HGDNs), and early HCC (eHCCs) to progressed HCCs (pHCCs) [10–15]. Despite evidence of the role of CSCs and tumor stroma in HCC, the development of CSCs, alterations in tumor stroma, and their relationship during multistep hepatocarcinogenesis have never been investigated. In this study, we focused on B viral multistep hepatocarcinogenesis, as it is the main etiology of HCC in Asia, including China and Korea [16]. We analyzed the stmeness features in combination with alterations in the tumoral stroma including CAFs, TAMs and IL-6-positive cells in B viral human multistep hepatocarcinogenesis including LGDNs, HGDNs, eHCCs, and pHCCs.

## Materials and Methods

### Liver tissue samples and pathological examination

The liver samples for this study were collected from Severance Hospital, Yonsei University Medical Center in Seoul. The samples comprised 36 LGDNs, 48 HGDNs, 30 eHCCs, and 51 pHCCs collected from 94 patients, including 72 men and 22 women, of ages ranging from 40 to 71 years (54.6 ± 7.22, mean ± standard deviation). Clinicopathological information for all patients is described in S1 Table. All cases were hepatitis B virus (HBV) related. Representative sections were submitted for routine histological examination. All nodular lesions were evaluated according to the criteria proposed by the International Consensus Group for Hepatocellular Neoplasia [15]. HCC differentiation was determined on the basis of Edmondson-Steiner grade [17].

The liver tissues were snap-frozen in liquid nitrogen and stored at -80°C. Fresh frozen liver specimens were provided by the Liver Cancer Specimen Bank, National Research Resource Bank program by the Korea Science and Engineering Foundation under the Ministry of Science and Technology. This study was approved by the Institutional Review Board of Severance Hospital, Yonsei University College of Medicine, and the need for patient consent was waived (2014-0253-004, Seoul, Korea).

### Total RNA extraction and Quantitative real-time PCR

Total RNA was extracted from 108 snap-frozen liver tissues using TRIzol (Invitrogen, Carlsbad, CA). The transcription into cDNA was synthesized using High Capacity RNA-to- cDNA kit according to the manufacturer’s instructions. PCR was performed from the total RNA to confirm the absence of genomic DNA contamination. PCR reactions were performed using gene-specific primers and probes with an ABI PRISM 7700 Sequence Detection System (Applied Biosystems, Foster City, CA) and software (Applied Biosystems) according to TaqMan protocols. Information on the primer/probe sets is summarized in S2 Table. Each reaction was performed in triplicate. A non-template reaction was included all experiments as a negative control.

### Immunohistochemistry

Immunohistochemical stains were performed for the antibodies described in S3 Table on available representative sections of formalin-fixed, paraffin-embedded tissues as follows: EpCAM and K19 in 35 LGDNs, 43 HGDNs, 27 eHCCs, and 51 pHCCs; α-smooth muscle actin (α-SMA), IL-6 and CD133 in 35 LGDNs, 41 HGDNs, 25 eHCCs, and 51 pHCCs; CD68 in 35 LGDNs, 42 HGDNs, 24 eHCCs and 51 pHCCs; CD163 in 34 LGDNs, 38 HGDNs, 20 eHCCs and 48 pHCCs. The number of cases varied due to the limited amount of tissue sections for some cases. Immunohistochemical stain was performed using an Envision kit (DAKO, Glostrup, Denmark) according to the manufacturer’s instructions.

The protein expression of EpCAM, K19, CD133, CD68, CD163 and IL-6 was interpreted in a semiquantitative manner. The expression of each marker was evaluated as positive when it was detected in more than 5% of tumor epithelial/stromal cells with moderate to strong intensity and was graded on a scale of 0-1-2-3 (0, <5% cells; 1, 5–10%; 2, 11–50%; 3, 51–100% of tumor epithelial/stromal cells). The expression of α-SMA (+) CAFs was evaluated semiquantitatively as follows: 0, no or few positive cells only identified upon careful examination under a high-power magnification; 1, scattered positive cells easily identified under a medium-power magnification; 2, scattered or clustering of positive cells apparent under a low-power magnification; and 3, large number of positive cells apparent under a low-power magnification [18].

## Results

### Progressive enrichment of stemness features in B viral multistep hepatocarcinogenesis

**Fig 1:**
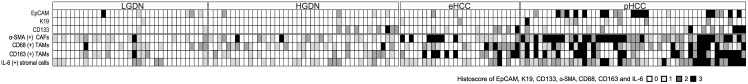
The heatmap presenting expression of EpCAM, K19, CD133, α-SMA (+) CAFs, CD68 (+) TAMs, CD163 (+) TAMs, and IL-6 (+) stromal cells in B viral multistep hepatocarcinogenesis. Histoscores of each marker are presented in the individual case of LGDNs, HGDNs, eHCCs and pHCCs.

**Fig 2:**
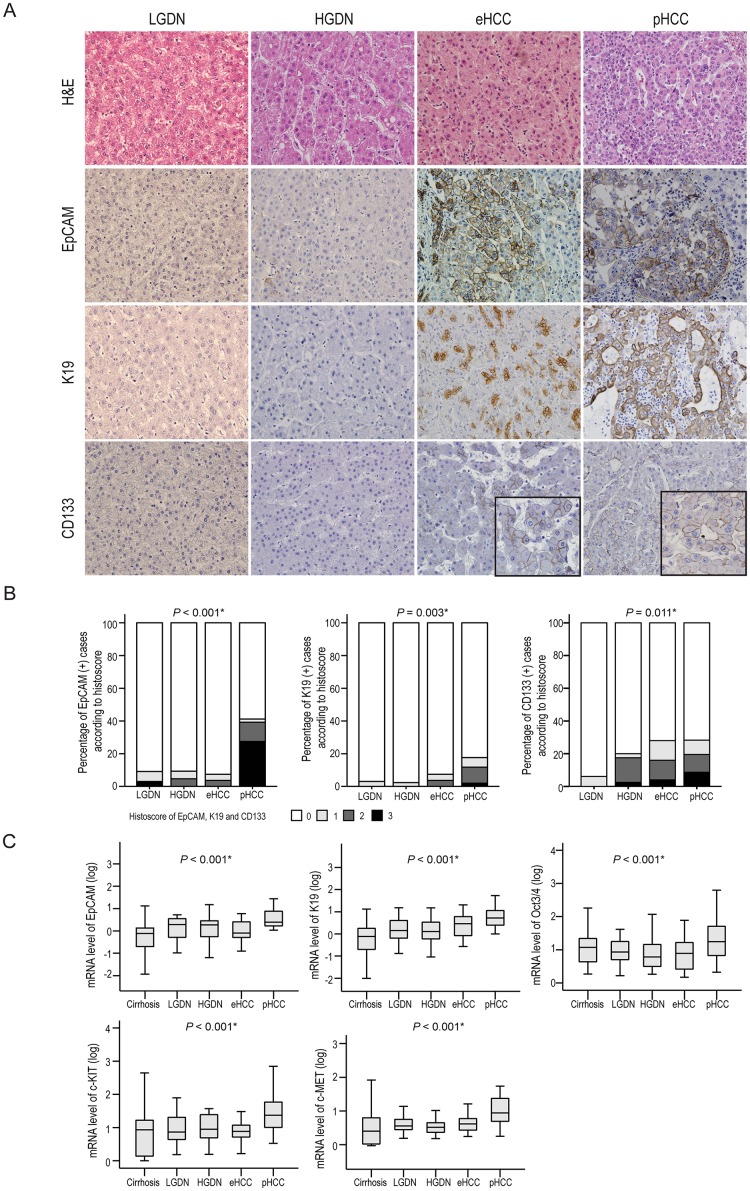
The expression of stemness features in B viral multistep hepatocarcinogenesis. A) Representative features of EpCAM, K19, and CD133 expression in LGDNs, HGDNs, eHCCs, and pHCCs are presented. Original magnification x200. Inset, high power magnification x400. B) Bar charts indicate the percentage of cases expressing EpCAM, K19, or CD133 protein in defined lesions of B viral multistep hepatocarcinogenesis. C) Box plots show the mRNA expression profiles of EpCAM, K19, Oct3/4, c-KIT, and c-MET in B viral multistep hepatocarcinogenesis. *Statistical significance (*P* <0.05).

The stemness features were evaluated by immunohistochemical staining for EpCAM, K19, and CD133 (Figs 1 and 2A). The protein expression levels of EpCAM, K19, and CD133 gradually increased with progression from LGDN, HGDN, and eHCC to pHCC with statistical significance: stemness markers were seen sporadically in DNs, more consistent in eHCC and peaking in pHCCs (*P* <0.05, for all; Fig 2B). The same trend was seen on analysis of mRNA levels for EpCAM, K19, Oct3/4, c-KIT, and c-MET (*P* <0.001, for all) (Fig 2C).

**Fig 3:**
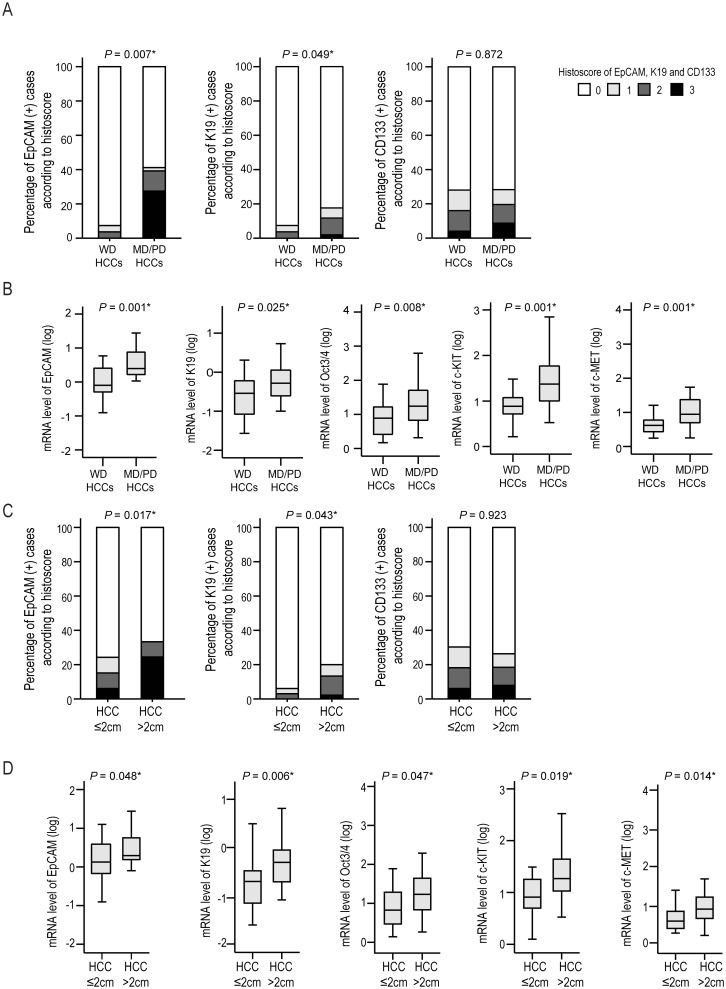
The expressions of stemness features according to differentiation (A, B) and size (C, D) of HCCs. Bar charts indicate the percentage of cases expressing EpCAM, K19 or CD133 protein. Box plots show the mRNA expression levels of EpCAM, K19, Oct3/4, c-KIT, and c-MET. *Statistical significance (*P* <0.05).

Among HCCs, EpCAM, and K19 were significantly enriched in moderately/poorly differentiated HCCs, compared to well differentiated HCCs (*P* = 0.007 and *P* = 0.049, respectively), while there was no significant difference in CD133 status according to differentiation (Fig 3A). EpCAM, K19, Oct3/4, c-KIT, and c-MET mRNA levels were also significantly higher in moderately/poorly differentiated HCCs than in well differentiated HCCs (Fig 3B). According to the size of HCCs, EpCAM and K19 protein expression levels were significantly higher in large HCCs (>2 cm) than in small HCCs (≤2 cm) (both, *P <*0.05) (Fig 3C), while there were no significant differences in CD133 status according to size. EpCAM, K19, Oct3/4, c-KIT, and c-MET mRNA levels were also significantly higher in larger HCCs (>2 cm) than in smaller HCCs (≤2 cm) (*P <*0.05 for all) (Fig 3D).

### Tumor stromal alterations and their relationship with stemness feature enrichment in B viral multistep hepatocarcinogenesis

**Fig 4:**
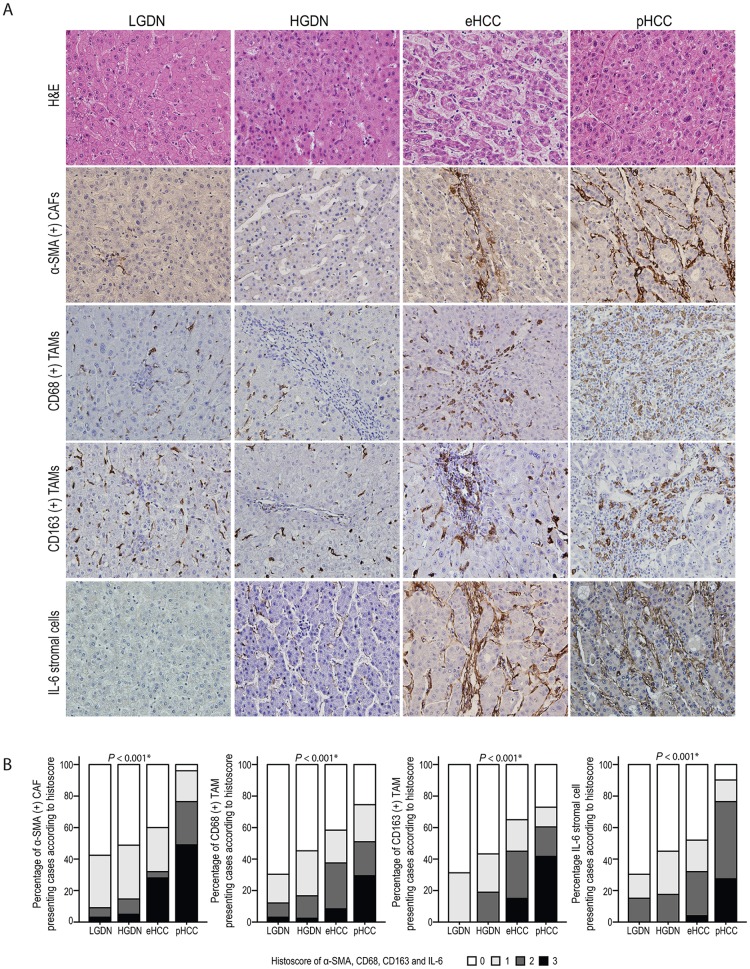
The alteration of tumor stromal cells in B viral multistep hepatocarcinogenesis. A) Representative features of α-SMA (+) CAFs, CD68 (+) TAMs, CD163 (+) TAMs, and IL-6 (+) stromal cells in LGDNs, HGDNs, eHCCs, and pHCCs are presented. Original magnification x200. B) Bar charts indicate the percentage of cases expressing α-SMA (+) CAFs, CD68 (+) TAMs, CD163 (+) TAMs, and IL-6 (+) stromal cells in B viral multistep hepatocarcinogenesis. *Statistical significance (*P* <0.05).

The tumor stromal cells expressing α-SMA, CD68, CD163, and IL-6 were evaluated in B viral multistep hepatocarcinogenesis (Figs 1 and 4A). There was a significant increase in the expression of α-SMA (+) CAFs with progression of multistep hepatocarcinogenesis (*P <*0.001): α-SMA (+) CAFs were discernible in small amounts in DNs, increased in eHCCs, and peaked in pHCCs. Regarding TAMs, the expression of CD68 (+) or CD163 (+) tumor stromal TAMs significantly increased with progression of multistep hepatocarcinogenesis (both, *P* <0.001) from LGDN to pHCC. IL-6 (+) tumor stromal cells also significantly increased with progression of multistep tumorigenesis (*P* <0.001) (Fig 4B).

**Fig 5:**
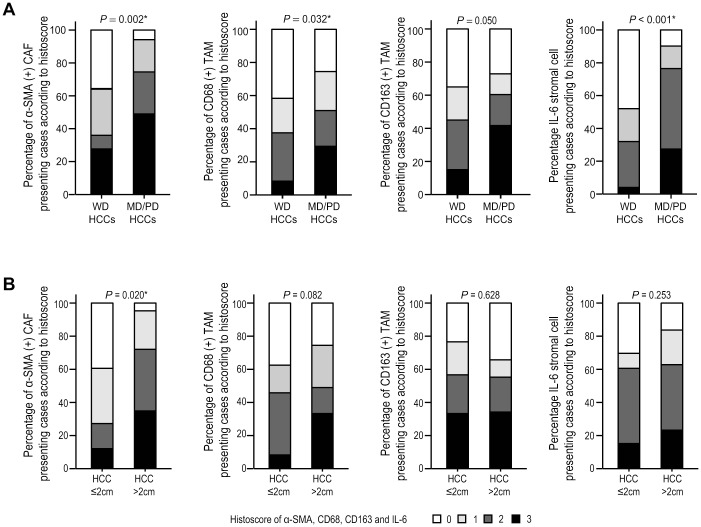
The alteration of tumor stromal cells according to differentiation and size of HCCs. Bar charts indicate the percentage of cases expressing α-SMA (+) CAFs, CD68 (+) TAMs, CD163 (+) TAMs, and IL-6 (+) stromal cells according to differentiation (A) and size (B) of HCCs. *Statistical significance (*P* <0.05).

Alterations of tumor stoma were also analyzed according to HCC differentiation and size. α-SMA (+) CAFs, CD68 (+) tumor stromal TAMs, and IL-6 (+) tumor stromal cells were significantly higher in moderately/poorly differentiated HCCs, compared to well differentiated HCCs (*P <*0.05 for all) (Fig 5A). α-SMA (+) CAFs were enriched in larger (>2 cm) HCCs, compared to smaller ones (≤2 cm) (*P* = 0.020) (Fig 5B).

**Fig 6:**
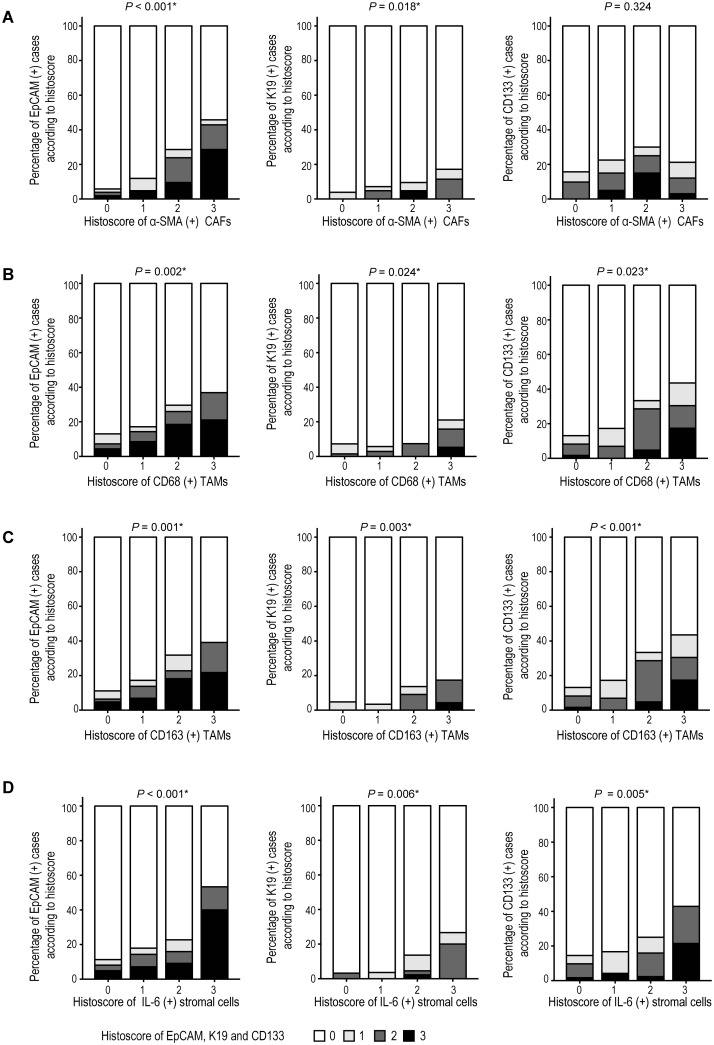
The correlation between enrichment of stemness features and tumor stromal alterations in B viral multistep hepatocarcinogenesis. Bar charts indicate the percentage of cases expressing EpCAM, K19, and CD133 protein expression according to the histoscore of CAFs (A), CD68 (+) TAMs (B), CD163 (+) TAMs (C), and IL-6 (+) stromal cells (D). *Statistical significance (*P* <0.05).

**Fig 7:**
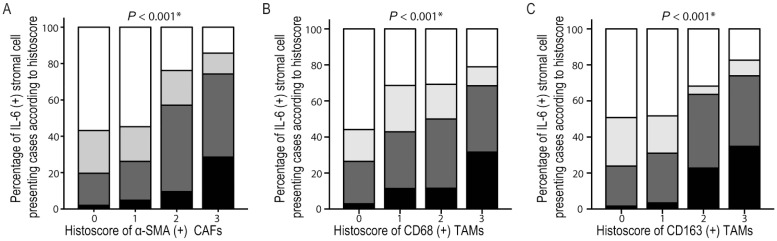
The correlation between IL-6 (+) stromal cells and CAFs or TAMs in B viral multistep hepatocarcinogenesis. IL-6 (+) stromal cells are well correlated with α-SMA (+) CAFs (A), CD68 (+) TAMs (B), and CD163 (+) TAMs (C). *Statistical significance (*P* <0.05).

The relation between tumor stromal alterations and stemness feature enrichment was evaluated in B viral multistep hepatocarcinogenesis. α-SMA (+) CAFs were enriched together with EpCAM and K19 expression (both, *P* <0.05), but not with CD133 (Fig 5A). CD68 (+) or CD163 (+) tumor stromal TAMs were all enriched together with EpCAM, K19 and CD133 expression (*P* <0.05 for all) (Fig 6B and 6C). IL-6 (+) tumor stromal cells were enriched together with EpCAM, K19 and CD133 expression (*P* <0.05 for all) (Fig 6D). IL-6 (+) tumor stromal cells also showed positive correlations with α-SMA (+) CAFs, CD68 (+) and CD163 (+) tumor stromal TAMs (Fig 7).

## Discussion

HCCs expressing stemness-related markers, such as K19, CD133, or EpCAM, are reportedly associated with a higher incidence of vascular invasion and a poorer prognosis, compared to HCCs that do not express these markers [2, 3]. However, whether CSCs (cancer stem cell features) are present in the early stages of human multistep hepatocarcinogenesis, including precancerous lesions, has yet to be clarified. In this study, we discovered a significant increase in the expression of stemness features with progression of B viral multistep hepatocarcinogenesis: cancer stemness features were seen sporadically in DNs, more consistent in eHCCs, and peaked in pHCCs. The expression levels of stemness features were also higher in less differentiated or larger HCCs. Taken together, these data suggest that the enrichment of stemness features takes place during B viral multistep hepatocarcinogenesis, with greater expansion in (more) progressed HCCs and thus in the more advanced phases of tumorigenesis. Recently, mature hepatocytes were found to exhibit unexpected plasticity upon direct de-differentiation to progenitor cells in culture [19], and complementary fate-tracing approaches that are able to label progenitor/biliary compartments and hepatocytes demonstrated that K19 and α-fetoprotein-positive cells within HCCs are hepatocyte-derived in murine hepatocarcinogenesis [20]. Similarly, K19 positivity was reported to be acquired with cancer progression in rat hepatocarcinogenesis [21]. Taken together, we speculate that CSCs of HCC are more likely (phenotypical features acquired from) to be derived from de-differentiated malignant hepatocytes that are selected and enriched during tumor progression. Although CSCs of HCC may possibly originate from pre-existing and transformed hepatic progenitor cells, this might be a minor pathway.

In this study, we also investigated alteration in the tumor stroma, including CAFs and TAMs, across B viral multistep hepatocarcinogenesis. α-SMA (+) CAFs were significantly increased during hepatocarcinogenesis, showing low levels in DNs, increasing in eHCC, and peaking in pHCCs. These changes were particularly evident in poorly differentiated and larger tumors. Macrophages can be sub-classified into classically (M1) and alternatively (M2)-activated macrophages. CD163 is a specific marker for the M2 phenotype, and CD68 is a marker for both M1 and M2 phenotypes. Interestingly, we found both of CD68 (+) and CD163 (+) TAMs gradually increased from DNs to incipient HCCs (eHCC), and peaked in pHCCs. CD68 (+) TAMs also enriched in moderately/poorly differentiated HCCs compared to well differentiated HCCs.

Tumor stroma regulates epithelial tumor cells via paracrine signaling [5]. In this study, IL-6 expression was mainly observed in tumoral stromal cells, and well correlated with those of α-SMA (+) CAFs, CD68 (+) and CD163 (+) TAMs. IL-6 (+) stromal cells were found in DNs at low levels and gradual increased with progression of multistep hepatocarcinogenesis; IL-6 (+) stromal cells showed the highest level in pHCCs. IL-6 (+) stromal cells were also higher in moderately/poorly differentiated HCCs compared to well differentiated HCCs. Taken together, the cancer niche of tumor stroma seems to undergo a dynamic modulation to become more tumor permissive during multistep hepatocarcinogenesis: first, by setting a precancerous niche in DNs as a necessary (early) step required for initiated cells to survive and evolve, and then a more mature and expanded niche that could accompany tumor promotion and progression into pHCCs.

Previously, implantation of HCC cells with hepatic stellate cells in nude mice was found to boost tumor growth and invasiveness, as well as to suppress tumor necrosis [22], and culturing HCC cells with LX2, a multipotent human hepatic stellate cell line, was reported to induce the production of pro-inflammatory cytokines including IL-6 that promoted HCC proliferation and migration through cross-talk with LX2 [23]. Interestingly, the present study revealed that the expression levels of HSC markers corresponded with changes in tumor stroma. EpCAM or K19 expression was well correlated with that of α-SMA (+) CAFs. EpCAM, K19 or CD133 expression also showed a significant correlation with those of tumor stromal CD68 (+)/CD163 (+) TAMs and IL-6 (+) stromal cells. All these data suggest that CSCs are not a fixed cell population, but rather are dynamic and likely regulated by tumor stromal factors. Our own data suggest that cross-talk and interactions between tumoral epithelial cells and progressively altered tumor stroma might facilitate the enrichment of CSCs (stemness-properties of tumor cells) with progression of multistep hepatocarcinogenesis. Accordingly, IL-6, secreted by CAFs and TMAs, was shown to promote the expansion of CSCs via STAT3 signaling and α-SMA (+) CAFs were reported to boost liver tumor-initiating cells through c-Met/FRA1/HEY1 signaling in *vitro* and animal study of HCCs [24, 25]. Interestingly enough CAFs, which in the present study were significantly enriched with IL-6-positive stromal cells particularly in advanced and poorly differentiated HCCs, have been recently shown to make the HCC microenvironment more tolerogenic, by inducing the generation of myeloid-derived suppressor cells through IL-6-mediated STAT3 activation. These data suggest that the “in situ” analysis of the interaction between hepatic cancer cells and those of the tumoral stroma like CAFs, might be of help in the future to discern HCC with distinct tolerogenic features, for potential therapeutic applications [26].

This study focused on B viral hepatocarcinogenesis. HBV is frequently integrated into host genomes, and nuclear HBV X protein (HBx) may be associated with hepatocellular transformation, affecting transcriptional machinery. Recently, HepG2 cells stably transduced with HBx showed increased EpCAM expression upon activation of β-catenin and epigenetic regulation of miR-181 [27], as well as decreased E-cadherin expression by hypermethylation of CDH1 [28]. Thus, HBx might be considered as an additional player in the promotion of a switch in gene expression to “stemness” in hepatocarcinogenesis. Further studies comparing the expression of CSC markers between B viral and C viral multistep hepatocarcinogenesis are required.

In conclusion, we have provided supportive evidence suggesting that B viral hepatocarcinogenesis is characterized by a progressive enrichment of stemness features by malignant hepatocytes, likely facilitated by the increasing interaction with tumor stromal cells including CAFs, TAMs, and IL-6 (+) cells. Therefore, in B viral hepatocarcinogenesis, interactions between CSCs and the tumoral stroma, although starting early, seem to play a major role in tumor progression. This combined morpho-phenotypic analysis of malignant epithelial cells and stromal cells in HCC illustrates a more comprehensive scenario of HCC setting and dynamics which may be forerunners of information for potential translational applications.

## Supporting Information

S1 Table Summary of clinicopathological information. (DOCX)

S2 Table Primer/probe sets used in this study. (DOCX)

S3 Table Antibodies used in this study. (DOCX)
