# Supplementary material for: Progressive Enrichment of Stemness Features and Tumor Stromal Alterations in Multistep Hepatocarcinogenesis
Source: PLoS One. 2017 Jan 23;12(1):e0170465. doi: 10.1371/journal.pone.0170465 (PMC5256873; doi:10.1371/journal.pone.0170465)
Supplement: S1 Table — (DOCX) [file pone.0170465.s001.docx]

**S1 Table. Summary of clinicopathological information**

| Patient No | Sex | Age | Etiology | Pathological diagnosis^a^ |
| --- | --- | --- | --- | --- |
| 1 | F | 60 | HBV | Cirrhosis, HGDN, HGDN, eHCC, pHCC-II |
| 2 | F | 54 | HBV | Cirrhosis, HGDN, pHCC-II |
| 3 | M | 59 | HBV | Cirrhosis, LGDN, HGDN |
| 4 | M | 48 | HBV | Cirrhosis, eHCC, pHCC-II, pHCC-III |
| 5 | F | 54 | HBV | Cirrhosis, HGDN |
| 6 | M | 50 | HBV | Cirrhosis, pHCC-II |
| 7 | M | 54 | HBV | Cirrhosis, HGDN |
| 8 | M | 60 | HBV | Cirrhosis, HGDN, eHCC, pHCC-II |
| 9 | M | 50 | HBV | Cirrhosis, eHCC |
| 10 | M | 54 | HBV | Cirrhosis, HGDN |
| 11 | M | 59 | HBV | Cirrhosis, HGDN |
| 12 | M | 50 | HBV | Cirrhosis, eHCC |
| 13 | M | 48 | HBV | Cirrhosis, eHCC |
| 14 | M | 54 | HBV | Cirrhosis, HGDN, HGDN, HGDN, HGDN, eHCC, pHCC-II |
| 15 | M | 54 | HBV | Cirrhosis, HGDN, HGDN, pHCC-II |
| 16 | M | 54 | HBV | Cirrhosis, HGDN, HGDN |
| 17 | M | 62 | HBV | Cirrhosis, eHCC, pHCC-II , pHCC-II , pHCC-III |
| 18 | F | 54 | HBV | Cirrhosis, LGDN |
| 19 | M | 56 | HBV | Cirrhosis, LGDN |
| 20 | M | 40 | HBV | Cirrhosis, pHCC-III |
| 21 | F | 50 | HBV | Cirrhosis, LGDN, LGDN, LGDN |
| 22 | M | 48 | HBV | Cirrhosis, eHCC, pHCC-II |
| 23 | M | 46 | HBV | Cirrhosis, pHCC-III |
| 24 | M | 42 | HBV | Cirrhosis, LGDN |
| 25 | M | 47 | HBV | Cirrhosis, eHCC, pHCC-II |
| 26 | M | 43 | HBV | Cirrhosis, LGDN, LGDN, pHCC-III |
| 27 | M | 57 | HBV | Cirrhosis, LGDN, LGDN, pHCC-II |
| 28 | M | 54 | HBV | Cirrhosis, LGDN, HGDN, eHCC, eHCC |
| 29 | F | 56 | HBV | Cirrhosis, LGDN, eHCC |
| 30 | F | 66 | HBV | Cirrhosis, pHCC-III |
| 31 | M | 41 | HBV | Cirrhosis, pHCC-II |
| 32 | M | 46 | HBV | Cirrhosis, pHCC-II |
| 33 | M | 59 | HBV | Cirrhosis, HGDN, eHCC, eHCC |
| 34 | M | 47 | HBV | Cirrhosis, pHCC-III |
| 35 | F | 58 | HBV | Cirrhosis, pHCC-II |
| 36 | M | 54 | HBV | Cirrhosis, pHCC-III |
| 37 | F | 42 | HBV | Cirrhosis, LGDN, HGDN, pHCC-III |
| 38 | F | 66 | HBV | Cirrhosis, pHCC-III |
| 39 | M | 64 | HBV | Cirrhosis, HGDN, eHCC |
| 40 | M | 47 | HBV | Cirrhosis, pHCC-III |
| 41 | M | 61 | HBV | Cirrhosis, LGDN, LGDN, LGDN, LGDN, LGDN, LGDN, HGDN, HGDN, HGDN, HGDN, HGDN, eHCC, eHCC |
| 42 | M | 45 | HBV | Cirrhosis, pHCC-II |
| 43 | M | 68 | HBV | Cirrhosis, LGDN, LGDN, pHCC-II , pHCC-II |
| 44 | M | 62 | HBV | Cirrhosis, LGDN, HGDN |
| 45 | M | 71 | HBV | Cirrhosis, pHCC-III |
| 46 | M | 56 | HBV | Cirrhosis, eHCC |
| 47 | M | 47 | HBV | Cirrhosis, LGDN, pHCC-III |
| 48 | M | 64 | HBV | Cirrhosis, pHCC-III |
| 49 | M | 51 | HBV | Cirrhosis, pHCC-III |
| 50 | M | 60 | HBV | Cirrhosis, LGDN , HGDN |
| 51 | M | 60 | HBV | Cirrhosis, pHCC-III |
| 52 | M | 48 | HBV | Cirrhosis, pHCC-III |
| 53 | M | 65 | HBV | Cirrhosis, pHCC-II |
| 54 | M | 47 | HBV | Cirrhosis, pHCC-II |
| 55 | M | 63 | HBV | Cirrhosis, HGDN |

**S1 Table. Continued**

| Patient No | Sex | Age | Etiology | Pathological diagnosis^a^ |
| --- | --- | --- | --- | --- |
| 56 | M | 61 | HBV | Cirrhosis, HGDN |
| 57 | F | 69 | HBV | Cirrhosis, LGDN, HGDN |
| 58 | M | 61 | HBV | Cirrhosis, LGDN |
| 59 | M | 52 | HBV | Cirrhosis, HGDN, eHCC |
| 60 | F | 53 | HBV | Cirrhosis, HGDN |
| 61 | F | 55 | HBV | Cirrhosis, LGDN, HGDN |
| 62 | M | 57 | HBV | Cirrhosis, LGDN |
| 63 | M | 57 | HBV | Cirrhosis, LGDN, pHCC-II |
| 64 | M | 54 | HBV | Cirrhosis, eHCC |
| 65 | M | 61 | HBV | Cirrhosis, LGDN, eHCC |
| 66 | F | 55 | HBV | Cirrhosis, eHCC |
| 67 | F | 64 | HBV | Cirrhosis, eHCC |
| 68 | M | 53 | HBV | Cirrhosis, HGDN |
| 69 | F | 53 | HBV | Cirrhosis, HGDN |
| 70 | F | 62 | HBV | Cirrhosis, HGDN, eHCC, pHCC-III |
| 71 | F | 66 | HBV | Cirrhosis, LGDN |
| 72 | F | 60 | HBV | Cirrhosis, HGDN, |
| 73 | M | 41 | HBV | Cirrhosis, eHCC, pHCC-II |
| 74 | M | 71 | HBV | Cirrhosis, LGDN, |
| 75 | M | 54 | HBV | Cirrhosis, HGDN, eHCC, pHCC-II |
| 76 | M | 51 | HBV | Cirrhosis, HGDN |
| 77 | M | 55 | HBV | Cirrhosis, HGDN, eHCC |
| 78 | M | 51 | HBV | Cirrhosis, HGDN, eHCC |
| 79 | F | 60 | HBV | Cirrhosis, HGDN |
| 80 | M | 49 | HBV | Cirrhosis, HGDN, pHCC-II |
| 81 | M | 47 | HBV | Cirrhosis, pHCC-II |
| 82 | M | 49 | HBV | Cirrhosis, eHCC |
| 83 | M | 52 | HBV | Cirrhosis, HGDN |
| 84 | M | 44 | HBV | Cirrhosis, LGDN, pHCC-II |
| 85 | M | 46 | HBV | Cirrhosis, LGDN |
| 86 | M | 49 | HBV | Cirrhosis, pHCC-III |
| 87 | M | 54 | HBV | Cirrhosis, pHCC-II |
| 88 | F | 55 | HBV | Cirrhosis, pHCC-II |
| 89 | M | 54 | HBV | Cirrhosis, LGDN, HGDN |
| 90 | F | 52 | HBV | Cirrhosis, pHCC-II |
| 91 | M | 47 | HBV | Cirrhosis, HGDN |
| 92 | M | 66 | HBV | Cirrhosis, pHCC-III |
| 93 | M | 59 | HBV | Cirrhosis, HGDN, pHCC-III |
| 94 | M | 62 | HBV | Cirrhosis, pHCC-II |

Abbreviations: M, male; F, female; HBV, hepatitis B virus; LGDN, low grade dysplastic nodule; HGDN, high grade dysplastic nodule; eHCC, early hepatocellular carcinoma; pHCC, progressed HCC.

^a^HCC grading according to the Edmondson and Steiner classification
