# Supplementary material for: Progressive Enrichment of Stemness Features and Tumor Stromal Alterations in Multistep Hepatocarcinogenesis
Source: PLoS One. 2017 Jan 23;12(1):e0170465. doi: 10.1371/journal.pone.0170465 (PMC5256873; doi:10.1371/journal.pone.0170465)
Supplement: S2 Table — (DOCX) [file pone.0170465.s002.docx]

**S2 Table. Primer/probe sets used in this study**

| Primer | The assay ID | Source |
| --- | --- | --- |
| EpCAM | Hs00901887_m1 | Applied Biosystems Inc.; Foster City, CA, USA |
| K19 | Hs00761767_s1 | Applied Biosystems Inc.; Foster City, CA, USA |
| Oct3/4 | Hs00999632_g1 | Applied Biosystems Inc.; Foster City, CA, USA |
| c-KIT | Hs00174029_m1 | Applied Biosystems Inc.; Foster City, CA, USA |
| c-MET | Hs01565582_g1 | Applied Biosystems Inc.; Foster City, CA, USA |
| GAPDH | Hs99999905_m1 | Applied Biosystems Inc.; Foster City, CA, USA |
