# Supplementary material for: Progressive Enrichment of Stemness Features and Tumor Stromal Alterations in Multistep Hepatocarcinogenesis
Source: PLoS One. 2017 Jan 23;12(1):e0170465. doi: 10.1371/journal.pone.0170465 (PMC5256873; doi:10.1371/journal.pone.0170465)
Supplement: S3 Table — (DOCX) [file pone.0170465.s003.docx]

**S3 Table. Antibodies used in this study**

| Antibody | Source | Clone | Dilution | Antigen retrieval |
| --- | --- | --- | --- | --- |
| EpCAM | Calbiochem (Darmstadt, Germany) | OP187 | 1:3000 | Microwave, citrate (pH 6.0) |
| K19 | DAKO (Glostrup, Denmark) | RCK108 | 1:100 | Enzyme (DAKO) |
| CD133 | Miltenyi Biotec (Teterow, Germany) | W6B3C1 | 1:25 | Microwave, citrate (pH 6.0) |
| α-SMA | DAKO (Glostrup, Denmark) | 1A4 | 1:3,000 | No treatment |
| CD68 | DAKO (Glostrup, Denmark) | KP1 | 1:300 | Microwave, citrate (pH 6.0) |
| CD163 | Cell Marque (Rocklin, CA, USA) | MRQ-26 | 1:50 | Microwave, citrate (pH 6.0) |
| IL-6 | Abcam, (Cambridge, UK) |  | 1:100 | Enzyme (DAKO) |

Abbreviations: CD, cluster of differentiation; α-SMA, α-smooth muscle actin; IL, interleukin; EpCAM, epithelial cell adhesion molecule; K, keratin
